# [(*Z*)-*O*-Ethyl-*N*-(*p*-tol­yl)thio­carbamato-κ*S*](triphenyl­phosphine)-κ*P*]gold(I)

**DOI:** 10.1107/S1600536809047618

**Published:** 2009-11-14

**Authors:** Primjira P. Tadbuppa, Edward R. T. Tiekink

**Affiliations:** a Department of Chemistry, National University of Singapore, Singapore 117543; b Department of Chemistry, University of Malaya, 50603 Kuala Lumpur, Malaysia

## Abstract

The title compound, [Au(C_10_H_12_NOS)(C_18_H_15_P)], features a linear *S*,*P*-donor set about the central Au atom. The thio­carbamate ligand is orientated to place the aryl ring in close proximity to Au [the closest Au⋯C distance is 3.238 (4) Å], which results in a small deviation from the ideal linear P—Au—S geometry.

## Related literature

For structural systematics and luminescence properties of phosphinegold(I) carbonimidothio­ates, see: Ho *et al.* (2006 ▶); Ho & Tiekink (2007 ▶); Kuan *et al.* (2008 ▶). For the synthesis, see Hall *et al.* (1993 ▶). For gold⋯π interactions, see: Tiekink & Zukerman-Schpector (2009 ▶).
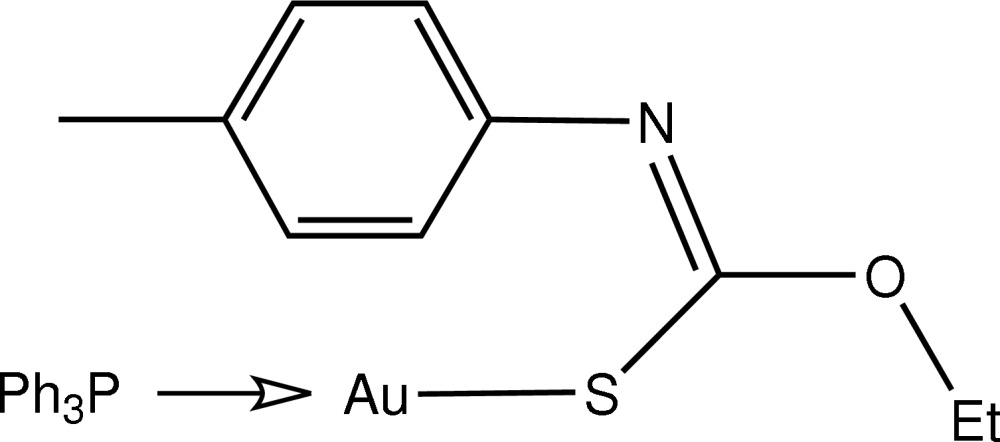


## Experimental

### 

#### Crystal data

[Au(C_10_H_12_NOS)(C_18_H_15_P)]
*M* *_r_* = 653.50
Triclinic, 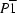

*a* = 8.6676 (5) Å
*b* = 12.1397 (6) Å
*c* = 13.2378 (7) Å
α = 65.482 (1)°
β = 89.765 (1)°
γ = 80.635 (1)°
*V* = 1247.30 (12) Å^3^
*Z* = 2
Mo *K*α radiation
μ = 6.07 mm^−1^
*T* = 223 K
0.31 × 0.16 × 0.16 mm

#### Data collection

Bruker SMART CCD diffractometer
Absorption correction: multi-scan (*SADABS*; Bruker, 2000 ▶) *T*_min_ = 0.311, *T*_max_ = 1
8852 measured reflections
5702 independent reflections
5380 reflections with *I* > 2σ(*I*)
*R*_int_ = 0.019

#### Refinement

*R*[*F* ^2^ > 2σ(*F* ^2^)] = 0.025
*wR*(*F* ^2^) = 0.074
*S* = 1.04
5702 reflections
299 parameters
H-atom parameters constrained
Δρ_max_ = 1.41 e Å^−3^
Δρ_min_ = −0.98 e Å^−3^

### 

Data collection: *SMART* (Bruker, 2000 ▶); cell refinement: *SAINT* (Bruker, 2000 ▶); data reduction: *SAINT*; program(s) used to solve structure: *PATTY* in *DIRDIF92* (Beurskens *et al.*, 1992 ▶); program(s) used to refine structure: *SHELXL97* (Sheldrick, 2008 ▶); molecular graphics: *DIAMOND* (Brandenburg, 2006 ▶); software used to prepare material for publication: *publCIF* (Westrip, 2009 ▶).

## Supplementary Material

Crystal structure: contains datablocks global, I. DOI: 10.1107/S1600536809047618/lh2951sup1.cif

Structure factors: contains datablocks I. DOI: 10.1107/S1600536809047618/lh2951Isup2.hkl

Additional supplementary materials:  crystallographic information; 3D view; checkCIF report

## Figures and Tables

**Table:** 

Au—S1	2.2964 (9)
Au—P1	2.2601 (9)

**Table:** 

P1—Au—S1	177.07 (3)

**Fig. 1.**
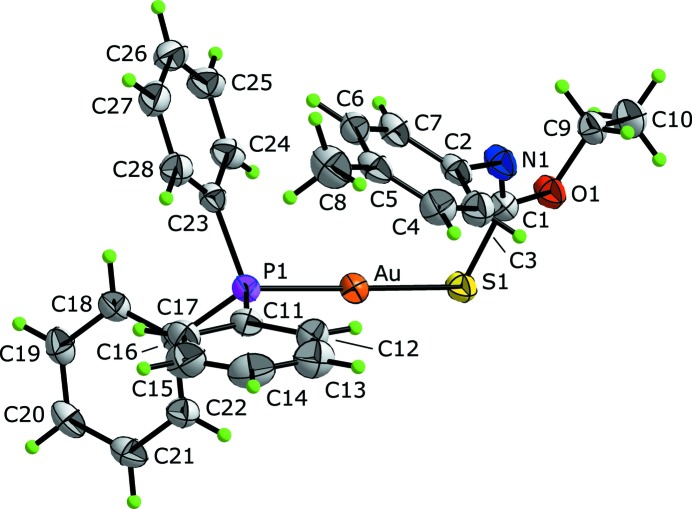
Molecular structure of (I) showing atom-labelling scheme and displacement ellipsoids at the 50% probability level.

## supplementary crystallographic information

### Comment

Phosphinegold(I) thiocarbamides have proved relatively easy to crystallize making systematic structural investigations possible, such as monitoring the influence of phosphine and/or thiocarbamato ligands upon supramolecular aggregation patterns (Ho & Tiekink, 2007; Kuan *et al.*, 2008) and luminescence (Ho *et al.* 2006). During these studies, the title compound, Ph_3_Au[SC(OEt)*N*(*p*-tolyl)], (I), was synthesized.

The gold atom in (I) exists in the expected linear geometry defined by a S,*P* donor set, Table 1 and Fig. 1. While the thiocarbamato anion shows the expected features, *i.e.* a *Z*-conformation about the C1-N1 bond and thiolate character [C1–S1 is 1.759 (3) Å and C1-N1 is 1.265 (5) Å], its orientation within the molecule is unusual. Normally in these type of phosphinegold(I) compounds, the orientation of the thiocarbamato ligand has the O1 atom in close proximity to the Au atom. However, in (I), the aryl ring is orientated towards Au [closest Au···C2 distance = 3.238 (4) Å, and Au···*Cg*(C2—C7) = 3.60 Å]. The close approach of the aryl ring is responsible for the small deviation from linearity of the S,*P* donor set, Table 1.

Electronic and steric effects have been cited as reasons for the variation in the coordination modes of thiocarbamato ligands in their phosphinegold(I) compounds, with N-bound *p*-tolyl groups known to promote Au···π interactions (Kuan *et al.*, 2008).

### Experimental

Compound (I) was prepared following the standard literature procedure from the reaction of Ph_3_AuCl and EtOC(*S*)*N*(*H*)(*p*-tolyl) in the presence of base (Hall *et al.*, 1993).

### Refinement

The H atoms were geometrically placed (C—H = 0.94–0.98 Å) and refined as riding with *U*_iso_(H) = 1.2–1.5*U*_eq_(C). The maximum and minimum residual electron density peaks of 1.41 and 0.98 e Å^-3^, respectively, were located 0.83 Å and 0.81 Å, respectively, from the Au atom.

### Crystal data

**Table:**

[Au(C_10_H_12_NOS)(C_18_H_15_P)]	*Z* = 2
*M**_r_* = 653.50	*F*(000) = 640
Triclinic, *P*1	*D*_x_ = 1.740 Mg m^−^^3^
Hall symbol: -P 1	Mo *K*α radiation, λ = 0.71069 Å
*a* = 8.6676 (5) Å	Cell parameters from 6912 reflections
*b* = 12.1397 (6) Å	θ = 2.4–29.9°
*c* = 13.2378 (7) Å	µ = 6.07 mm^−^^1^
α = 65.482 (1)°	*T* = 223 K
β = 89.765 (1)°	Block, colourless
γ = 80.635 (1)°	0.31 × 0.16 × 0.16 mm
*V* = 1247.30 (12) Å^3^	

### Data collection

**Table:**

Bruker SMART CCD diffractometer	5702 independent reflections
Radiation source: fine-focus sealed tube	5380 reflections with *I* > 2σ(*I*)
graphite	*R*_int_ = 0.019
ω scans	θ_max_ = 27.5°, θ_min_ = 1.9°
Absorption correction: multi-scan (*SADABS*; Bruker, 2000)	*h* = −11→11
*T*_min_ = 0.311, *T*_max_ = 1	*k* = −14→15
8852 measured reflections	*l* = −11→17

### Refinement

**Table:**

Refinement on *F*^2^	Primary atom site location: structure-invariant direct methods
Least-squares matrix: full	Secondary atom site location: difference Fourier map
*R*[*F*^2^ > 2σ(*F*^2^)] = 0.025	Hydrogen site location: inferred from neighbouring sites
*wR*(*F*^2^) = 0.074	H-atom parameters constrained
*S* = 1.04	*w* = 1/[σ^2^(*F*_o_^2^) + (0.0515*P*)^2^ + 0.0366*P*] where *P* = (*F*_o_^2^ + 2*F*_c_^2^)/3
5702 reflections	(Δ/σ)_max_ = 0.001
299 parameters	Δρ_max_ = 1.41 e Å^−^^3^
0 restraints	Δρ_min_ = −0.98 e Å^−^^3^

### Special details

**Table:**

Geometry. All s.u.'s (except the s.u. in the dihedral angle between two l.s. planes) are estimated using the full covariance matrix. The cell s.u.'s are taken into account individually in the estimation of s.u.'s in distances, angles and torsion angles; correlations between s.u.'s in cell parameters are only used when they are defined by crystal symmetry. An approximate (isotropic) treatment of cell s.u.'s is used for estimating s.u.'s involving l.s. planes.
Refinement. Refinement of *F*^2^ against ALL reflections. The weighted *R*-factor *wR* and goodness of fit *S* are based on *F*^2^, conventional *R*-factors *R* are based on *F*, with *F* set to zero for negative *F*^2^. The threshold expression of *F*^2^ > 2σ(*F*^2^) is used only for calculating *R*-factors(gt) *etc*. and is not relevant to the choice of reflections for refinement. *R*-factors based on *F*^2^ are statistically about twice as large as those based on *F*, and *R*- factors based on ALL data will be even larger.

### Fractional atomic coordinates and isotropic or equivalent isotropic displacement parameters (Å^2^)

**Table:**

	*x*	*y*	*z*	*U*_iso_*/*U*_eq_	
Au	0.133778 (13)	0.439781 (10)	0.172306 (9)	0.03279 (6)	
S1	−0.03428 (11)	0.61652 (8)	0.06097 (8)	0.03801 (18)	
P1	0.28847 (10)	0.26188 (8)	0.28627 (7)	0.03056 (17)	
O1	−0.1531 (3)	0.8119 (2)	0.0773 (2)	0.0404 (5)	
N1	0.0908 (4)	0.7459 (3)	0.1633 (3)	0.0434 (7)	
C1	−0.0164 (4)	0.7286 (3)	0.1099 (3)	0.0341 (6)	
C2	0.2387 (4)	0.6696 (3)	0.2015 (3)	0.0368 (7)	
C3	0.3647 (5)	0.6845 (4)	0.1339 (4)	0.0471 (8)	
H3	0.3482	0.7387	0.0581	0.056*	
C4	0.5130 (5)	0.6205 (4)	0.1775 (4)	0.0473 (9)	
H4	0.5966	0.6326	0.1308	0.057*	
C5	0.5420 (4)	0.5380 (3)	0.2896 (3)	0.0405 (8)	
C6	0.4168 (5)	0.5244 (3)	0.3562 (3)	0.0408 (8)	
H6	0.4331	0.4705	0.4322	0.049*	
C7	0.2677 (4)	0.5889 (3)	0.3127 (3)	0.0400 (8)	
H7	0.1844	0.5777	0.3596	0.048*	
C8	0.7037 (5)	0.4652 (4)	0.3367 (4)	0.0589 (11)	
H8A	0.7252	0.4645	0.4089	0.088*	
H8B	0.7810	0.5029	0.2867	0.088*	
H8C	0.7089	0.3815	0.3449	0.088*	
C9	−0.1632 (4)	0.9157 (3)	0.1064 (3)	0.0400 (7)	
H9A	−0.0921	0.9706	0.0639	0.048*	
H9B	−0.1352	0.8872	0.1859	0.048*	
C10	−0.3300 (5)	0.9812 (4)	0.0780 (4)	0.0523 (10)	
H10A	−0.3432	1.0516	0.0964	0.078*	
H10B	−0.3988	0.9256	0.1203	0.078*	
H10C	−0.3558	1.0090	−0.0009	0.078*	
C11	0.4880 (4)	0.2449 (3)	0.2467 (3)	0.0338 (6)	
C12	0.5368 (4)	0.3463 (3)	0.1625 (3)	0.0399 (7)	
H12	0.4657	0.4204	0.1259	0.048*	
C13	0.6905 (5)	0.3367 (4)	0.1334 (4)	0.0540 (10)	
H13	0.7241	0.4050	0.0778	0.065*	
C14	0.7948 (5)	0.2275 (5)	0.1856 (4)	0.0569 (11)	
H14	0.8991	0.2221	0.1658	0.068*	
C15	0.7465 (5)	0.1256 (4)	0.2670 (4)	0.0559 (11)	
H15	0.8175	0.0512	0.3019	0.067*	
C16	0.5923 (5)	0.1342 (4)	0.2969 (3)	0.0439 (8)	
H16	0.5587	0.0651	0.3512	0.053*	
C17	0.2276 (4)	0.1190 (3)	0.3033 (3)	0.0321 (6)	
C18	0.1892 (4)	0.0357 (3)	0.4049 (3)	0.0383 (7)	
H18	0.1924	0.0535	0.4675	0.046*	
C19	0.1460 (5)	−0.0739 (3)	0.4155 (4)	0.0444 (8)	
H19	0.1196	−0.1299	0.4846	0.053*	
C20	0.1424 (4)	−0.0992 (3)	0.3228 (4)	0.0456 (8)	
H20	0.1145	−0.1733	0.3291	0.055*	
C21	0.1794 (5)	−0.0163 (4)	0.2211 (4)	0.0453 (8)	
H21	0.1759	−0.0344	0.1587	0.054*	
C22	0.2216 (4)	0.0927 (3)	0.2101 (3)	0.0400 (8)	
H22	0.2461	0.1490	0.1404	0.048*	
C23	0.2960 (4)	0.2546 (3)	0.4263 (3)	0.0329 (6)	
C24	0.1596 (4)	0.3020 (3)	0.4609 (3)	0.0400 (7)	
H24	0.0671	0.3331	0.4138	0.048*	
C25	0.1605 (5)	0.3033 (4)	0.5653 (3)	0.0483 (9)	
H25	0.0678	0.3341	0.5892	0.058*	
C26	0.2956 (5)	0.2601 (4)	0.6341 (3)	0.0496 (9)	
H26	0.2960	0.2643	0.7034	0.059*	
C27	0.4302 (6)	0.2107 (4)	0.6016 (3)	0.0505 (9)	
H27	0.5214	0.1779	0.6500	0.061*	
C28	0.4315 (4)	0.2093 (3)	0.4973 (3)	0.0421 (8)	
H28	0.5245	0.1774	0.4745	0.051*	

### Atomic displacement parameters (Å^2^)

**Table:**

	*U*^11^	*U*^22^	*U*^33^	*U*^12^	*U*^13^	*U*^23^
Au	0.03425 (8)	0.02878 (8)	0.03499 (9)	−0.00471 (5)	0.00208 (5)	−0.01337 (6)
S1	0.0414 (4)	0.0333 (4)	0.0401 (4)	−0.0021 (3)	−0.0074 (3)	−0.0178 (4)
P1	0.0324 (4)	0.0270 (4)	0.0332 (4)	−0.0056 (3)	0.0028 (3)	−0.0134 (3)
O1	0.0382 (12)	0.0349 (12)	0.0474 (14)	0.0010 (10)	−0.0073 (10)	−0.0193 (11)
N1	0.0396 (16)	0.0362 (15)	0.0541 (19)	0.0010 (13)	−0.0107 (14)	−0.0214 (14)
C1	0.0376 (16)	0.0299 (15)	0.0355 (16)	−0.0050 (13)	0.0019 (13)	−0.0147 (13)
C2	0.0347 (16)	0.0320 (16)	0.0493 (19)	−0.0045 (13)	−0.0058 (14)	−0.0229 (15)
C3	0.048 (2)	0.0419 (19)	0.048 (2)	−0.0037 (16)	−0.0011 (17)	−0.0176 (17)
C4	0.042 (2)	0.048 (2)	0.053 (2)	−0.0086 (17)	0.0077 (17)	−0.0216 (18)
C5	0.0341 (16)	0.0389 (18)	0.056 (2)	−0.0046 (14)	−0.0038 (15)	−0.0279 (17)
C6	0.0454 (19)	0.0317 (16)	0.0439 (19)	−0.0042 (15)	−0.0082 (15)	−0.0153 (15)
C7	0.0358 (17)	0.0353 (17)	0.051 (2)	−0.0082 (14)	0.0031 (15)	−0.0194 (16)
C8	0.041 (2)	0.058 (3)	0.080 (3)	0.0024 (19)	−0.013 (2)	−0.034 (2)
C9	0.0420 (18)	0.0328 (16)	0.0432 (19)	0.0000 (14)	0.0002 (14)	−0.0162 (15)
C10	0.047 (2)	0.046 (2)	0.056 (2)	0.0086 (17)	−0.0033 (18)	−0.0202 (19)
C11	0.0319 (15)	0.0360 (16)	0.0370 (16)	−0.0052 (13)	0.0041 (12)	−0.0190 (14)
C12	0.0380 (17)	0.0413 (18)	0.0412 (18)	−0.0096 (14)	0.0066 (14)	−0.0173 (15)
C13	0.046 (2)	0.065 (3)	0.059 (3)	−0.0207 (19)	0.0164 (19)	−0.029 (2)
C14	0.039 (2)	0.082 (3)	0.059 (3)	−0.010 (2)	0.0087 (19)	−0.039 (3)
C15	0.046 (2)	0.066 (3)	0.053 (2)	0.009 (2)	0.0006 (18)	−0.028 (2)
C16	0.045 (2)	0.0393 (19)	0.045 (2)	−0.0010 (15)	0.0049 (16)	−0.0185 (16)
C17	0.0294 (14)	0.0291 (15)	0.0396 (17)	−0.0053 (12)	−0.0002 (12)	−0.0161 (13)
C18	0.0412 (17)	0.0340 (17)	0.0427 (18)	−0.0081 (14)	0.0037 (14)	−0.0186 (15)
C19	0.045 (2)	0.0335 (17)	0.051 (2)	−0.0118 (15)	0.0093 (16)	−0.0134 (16)
C20	0.0419 (19)	0.0358 (18)	0.067 (2)	−0.0097 (15)	0.0022 (17)	−0.0280 (18)
C21	0.047 (2)	0.047 (2)	0.053 (2)	−0.0094 (16)	−0.0005 (16)	−0.0313 (18)
C22	0.0443 (19)	0.0389 (18)	0.0398 (18)	−0.0078 (15)	−0.0010 (15)	−0.0191 (15)
C23	0.0380 (16)	0.0286 (15)	0.0352 (16)	−0.0098 (12)	0.0043 (13)	−0.0149 (13)
C24	0.0390 (17)	0.0413 (18)	0.0453 (19)	−0.0116 (14)	0.0076 (14)	−0.0222 (16)
C25	0.054 (2)	0.052 (2)	0.049 (2)	−0.0184 (18)	0.0194 (18)	−0.0282 (19)
C26	0.071 (3)	0.050 (2)	0.0357 (18)	−0.026 (2)	0.0125 (18)	−0.0202 (17)
C27	0.065 (3)	0.041 (2)	0.0396 (19)	−0.0112 (18)	−0.0068 (17)	−0.0106 (16)
C28	0.0433 (19)	0.0403 (18)	0.0423 (19)	−0.0064 (15)	0.0006 (15)	−0.0171 (15)

### Geometric parameters (Å, °)

**Table:**

Au—S1	2.2964 (9)	C12—C13	1.385 (5)
Au—P1	2.2601 (9)	C12—H12	0.9400
S1—C1	1.759 (3)	C13—C14	1.381 (7)
P1—C11	1.810 (3)	C13—H13	0.9400
P1—C17	1.819 (3)	C14—C15	1.387 (7)
P1—C23	1.820 (3)	C14—H14	0.9400
O1—C1	1.365 (4)	C15—C16	1.392 (6)
O1—C9	1.453 (4)	C15—H15	0.9400
N1—C1	1.265 (5)	C16—H16	0.9400
N1—C2	1.409 (4)	C17—C18	1.386 (5)
C2—C7	1.385 (5)	C17—C22	1.400 (5)
C2—C3	1.395 (5)	C18—C19	1.392 (5)
C3—C4	1.378 (6)	C18—H18	0.9400
C3—H3	0.9400	C19—C20	1.386 (6)
C4—C5	1.400 (6)	C19—H19	0.9400
C4—H4	0.9400	C20—C21	1.380 (6)
C5—C6	1.383 (6)	C20—H20	0.9400
C5—C8	1.510 (5)	C21—C22	1.380 (5)
C6—C7	1.385 (5)	C21—H21	0.9400
C6—H6	0.9400	C22—H22	0.9400
C7—H7	0.9400	C23—C24	1.390 (5)
C8—H8A	0.9700	C23—C28	1.391 (5)
C8—H8B	0.9700	C24—C25	1.389 (5)
C8—H8C	0.9700	C24—H24	0.9400
C9—C10	1.498 (5)	C25—C26	1.374 (6)
C9—H9A	0.9800	C25—H25	0.9400
C9—H9B	0.9800	C26—C27	1.374 (6)
C10—H10A	0.9700	C26—H26	0.9400
C10—H10B	0.9700	C27—C28	1.388 (6)
C10—H10C	0.9700	C27—H27	0.9400
C11—C16	1.390 (5)	C28—H28	0.9400
C11—C12	1.401 (5)		
			
P1—Au—S1	177.07 (3)	C13—C12—C11	119.6 (4)
C1—S1—Au	107.44 (12)	C13—C12—H12	120.2
C11—P1—C17	103.77 (15)	C11—C12—H12	120.2
C11—P1—C23	107.57 (15)	C14—C13—C12	120.4 (4)
C17—P1—C23	104.35 (15)	C14—C13—H13	119.8
C11—P1—Au	114.08 (12)	C12—C13—H13	119.8
C17—P1—Au	117.45 (11)	C13—C14—C15	120.4 (4)
C23—P1—Au	108.81 (11)	C13—C14—H14	119.8
C1—O1—C9	116.6 (3)	C15—C14—H14	119.8
C1—N1—C2	125.5 (3)	C14—C15—C16	119.7 (4)
N1—C1—O1	118.6 (3)	C14—C15—H15	120.2
N1—C1—S1	134.4 (3)	C16—C15—H15	120.2
O1—C1—S1	107.0 (2)	C11—C16—C15	120.1 (4)
C7—C2—C3	118.0 (3)	C11—C16—H16	119.9
C7—C2—N1	119.7 (3)	C15—C16—H16	119.9
C3—C2—N1	121.7 (3)	C18—C17—C22	119.4 (3)
C4—C3—C2	120.6 (4)	C18—C17—P1	122.3 (3)
C4—C3—H3	119.7	C22—C17—P1	118.3 (3)
C2—C3—H3	119.7	C17—C18—C19	120.9 (3)
C3—C4—C5	121.4 (4)	C17—C18—H18	119.6
C3—C4—H4	119.3	C19—C18—H18	119.6
C5—C4—H4	119.3	C20—C19—C18	119.0 (4)
C6—C5—C4	117.6 (3)	C20—C19—H19	120.5
C6—C5—C8	120.9 (4)	C18—C19—H19	120.5
C4—C5—C8	121.5 (4)	C21—C20—C19	120.5 (3)
C5—C6—C7	121.0 (4)	C21—C20—H20	119.8
C5—C6—H6	119.5	C19—C20—H20	119.8
C7—C6—H6	119.5	C20—C21—C22	120.7 (4)
C2—C7—C6	121.4 (4)	C20—C21—H21	119.6
C2—C7—H7	119.3	C22—C21—H21	119.6
C6—C7—H7	119.3	C21—C22—C17	119.5 (4)
C5—C8—H8A	109.5	C21—C22—H22	120.2
C5—C8—H8B	109.5	C17—C22—H22	120.2
H8A—C8—H8B	109.5	C24—C23—C28	119.1 (3)
C5—C8—H8C	109.5	C24—C23—P1	117.3 (3)
H8A—C8—H8C	109.5	C28—C23—P1	123.5 (3)
H8B—C8—H8C	109.5	C25—C24—C23	119.7 (4)
O1—C9—C10	106.2 (3)	C25—C24—H24	120.2
O1—C9—H9A	110.5	C23—C24—H24	120.2
C10—C9—H9A	110.5	C26—C25—C24	120.7 (4)
O1—C9—H9B	110.5	C26—C25—H25	119.7
C10—C9—H9B	110.5	C24—C25—H25	119.7
H9A—C9—H9B	108.7	C27—C26—C25	120.0 (4)
C9—C10—H10A	109.5	C27—C26—H26	120.0
C9—C10—H10B	109.5	C25—C26—H26	120.0
H10A—C10—H10B	109.5	C26—C27—C28	120.0 (4)
C9—C10—H10C	109.5	C26—C27—H27	120.0
H10A—C10—H10C	109.5	C28—C27—H27	120.0
H10B—C10—H10C	109.5	C27—C28—C23	120.4 (4)
C16—C11—C12	119.7 (3)	C27—C28—H28	119.8
C16—C11—P1	121.7 (3)	C23—C28—H28	119.8
C12—C11—P1	118.6 (3)		
			
C2—N1—C1—O1	−179.2 (3)	P1—C11—C16—C15	−177.8 (3)
C2—N1—C1—S1	−0.6 (7)	C14—C15—C16—C11	−1.1 (6)
C9—O1—C1—N1	0.6 (5)	C11—P1—C17—C18	116.3 (3)
C9—O1—C1—S1	−178.4 (2)	C23—P1—C17—C18	3.7 (3)
Au—S1—C1—N1	25.4 (4)	Au—P1—C17—C18	−116.8 (3)
Au—S1—C1—O1	−155.85 (19)	C11—P1—C17—C22	−62.9 (3)
C1—N1—C2—C7	−102.7 (5)	C23—P1—C17—C22	−175.5 (3)
C1—N1—C2—C3	86.4 (5)	Au—P1—C17—C22	64.0 (3)
C7—C2—C3—C4	−0.1 (6)	C22—C17—C18—C19	0.5 (5)
N1—C2—C3—C4	171.0 (4)	P1—C17—C18—C19	−178.8 (3)
C2—C3—C4—C5	0.8 (6)	C17—C18—C19—C20	0.3 (6)
C3—C4—C5—C6	−1.2 (6)	C18—C19—C20—C21	−0.7 (6)
C3—C4—C5—C8	178.3 (4)	C19—C20—C21—C22	0.4 (6)
C4—C5—C6—C7	1.0 (6)	C20—C21—C22—C17	0.4 (6)
C8—C5—C6—C7	−178.5 (4)	C18—C17—C22—C21	−0.8 (5)
C3—C2—C7—C6	−0.1 (5)	P1—C17—C22—C21	178.5 (3)
N1—C2—C7—C6	−171.4 (4)	C11—P1—C23—C24	160.8 (3)
C5—C6—C7—C2	−0.4 (6)	C17—P1—C23—C24	−89.4 (3)
C1—O1—C9—C10	−171.3 (3)	Au—P1—C23—C24	36.7 (3)
C17—P1—C11—C16	−41.1 (3)	C11—P1—C23—C28	−16.4 (3)
C23—P1—C11—C16	69.1 (3)	C17—P1—C23—C28	93.4 (3)
Au—P1—C11—C16	−170.1 (3)	Au—P1—C23—C28	−140.5 (3)
C17—P1—C11—C12	138.3 (3)	C28—C23—C24—C25	−0.1 (5)
C23—P1—C11—C12	−111.5 (3)	P1—C23—C24—C25	−177.4 (3)
Au—P1—C11—C12	9.3 (3)	C23—C24—C25—C26	1.1 (6)
C16—C11—C12—C13	−2.7 (5)	C24—C25—C26—C27	−2.4 (6)
P1—C11—C12—C13	177.8 (3)	C25—C26—C27—C28	2.7 (6)
C11—C12—C13—C14	1.0 (6)	C26—C27—C28—C23	−1.7 (6)
C12—C13—C14—C15	0.6 (7)	C24—C23—C28—C27	0.4 (5)
C13—C14—C15—C16	−0.6 (7)	P1—C23—C28—C27	177.6 (3)
C12—C11—C16—C15	2.7 (6)		
